# Specific and quantitative labeling of biomolecules using click chemistry

**DOI:** 10.3389/fphys.2014.00457

**Published:** 2014-11-24

**Authors:** Kenichi Horisawa

**Affiliations:** Division of Organogenesis and Regeneration, Department of Molecular and Cellular Biology, Medical Institute of Bioregulation, Kyushu UniversityFukuoka, Japan

**Keywords:** click chemistry, fluorescent labeling, metabolic labeling, bioorthogonal reaction

## Abstract

Specific and highly efficient fluorescent labeling techniques for biomolecules, especially for proteins, are required for the quantitative analyses of bio-phenomena and for subsequent systems biology. Although expression of exogenous proteins fused with fluorescent tags, such as green fluorescent protein, is the most widely used method for quantitative bio-analysis, the following problems need to be considered carefully: (1) precise stoichiometric control in living cells is difficult, and (2) the bulkiness of the fluorescent tags restricts analysis of the inherent physical and biological properties of the proteins. Therefore, novel techniques to specifically and stoichiometrically label intrinsic proteins or other biomolecules in living cells should be developed. Click chemistry reactions (e.g., Huisgen cycloaddition and Staudinger ligation) are the most promising approaches for this purpose, because these chemical reactions have following advantages: (1) bioorthogonal reactions; (2) mild reaction conditions suitable for fragile biomolecules, cells, and tissues; (3) extremely high reaction ratio; (4) small size of the functional groups for the cross-coupling reactions; (5) stable covalent bonding; and (6) simple metabolic labeling procedures in living cells, using various biomolecular analogs. Diverse quantitative biological studies have been carried out using this technology (e.g., quantification of novel synthesized proteins and observation of post-translational modifications). In this review, I explain the basics of chemical probing with click chemistry, and discuss its recent applications in the field of quantitative biology. Furthermore, I discuss the capability, significance, and future of the chemical probing of proteins, with an emphasis on the use of click chemistry in the field of the quantitative biology.

## Quantitative fluorescence imaging of biomolecules in living cells

Fluorescent imaging tools such as fluorescent microscopy are one of the most efficient and widely used modern techniques in the life sciences for analyzing the quantitative behavior of biomolecules in living cells, tissues, and organisms (Stephens and Allan, 2003). Although a lot of alternative imaging technologies exist, such as electron microscopy, autoradiography, and immunochemistry, the fluorescent labeling of biomolecules and their subsequent observation with various optical instruments shows greater advantages, especially in the area of high temporal resolution, as this is one of the most important factors that needs to be analyzed for understanding biomolecular dynamics. Around the end of the last century and at the beginning of this century, the application of green fluorescent protein (Shimomura et al., 1962; Xue et al., 1993; Chalfie et al., 1994; Heim and Tsien, 1996) and its derivatives (Shaner et al., 2005) drastically improved the available fluorescence imaging methods for live samples. The huge impact of genetic fluorescent labeling in living cells can be seen from the wide variety of available fluorescent proteins. Indeed, the Nobel prize was awarded to Drs. Shimomura, Chalfie, and Tsien, in 2008 for the discovery and application of green fluorescent protein.

An alternative to fluorescent protein tagging is the use of fluorescent chemical compounds. These chemicals have long been employed in the field of bioscience. Table 1 shows the difference in the properties of fluorescent proteins and chemicals. As live imaging tools, fluorescent proteins are more predominantly used as compared to their chemical counterparts, because the genetic labeling procedure is very easy and reliable. However, fluorescent proteins also show certain disadvantages, especially during quantitative analysis (Table 1). The biggest issue of the protein-tagging method is the difficulty in controlling the stoichiometry of the target proteins inside the cell. Basically, the number of the proteins is controlled at the transcriptional level in living cells. Although the transcriptional activity can be roughly regulated through an appropriate choice of promoters or other artificial molecular systems, strict stoichiometric control is beyond the capability of the current molecular biological technology. The second problem pertains to the adverse effects of the tagged fluorescent proteins. The molecular size of the fluorescent proteins is larger than that of the fluorescent chemicals and other widely used conventional protein tags (e.g., FLAG, HA, V5, T7, and Myc tags). The bulkiness of the fluorescent protein tag is likely to affect the behavior, stability, and function of the target proteins.

**Table 1:**
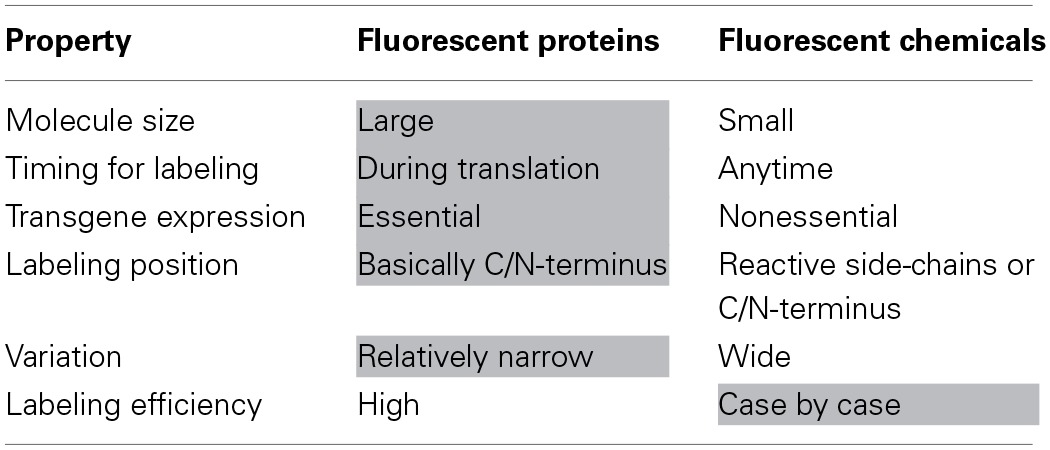
**Comparison of fluorescent proteins and chemicals**.

^*^Gray-shaded columns highlight the disadvantages for live cell imaging.

For the reasons stated above, caution must be exercised when using fluorescent proteins for quantitative analyses. On the other hand, some fluorescent chemicals exhibit properties appropriate for the quantitative fluorescent observation of biomolecules, including small molecule size, various labeling positions, nonessentiality of transgene expression, and wide variation of their optical spectrum (Jung et al., 2013). However, the efficient and specific conjugation of fluorescent chemicals to the target proteins poses a major problem. For example, the NHS ester and isothiocyanate coupling reactions are the most general methods for the fluorescent labeling of proteins, although their target functional (amino) group is present not only in the target protein but also in other biomolecules such as DNA and RNA. Therefore, this makes it impossible to label target molecules specifically if presented with a mixture of biomolecules.

Click chemistry reactions have been recently developed for chemical coupling. This chemical method has the potential to be a breakthrough in the field of live fluorescence imaging. In the following sections, I introduce some of the applications of click chemistry for cell and tissue imaging, and discuss the various applications of this technology in the field of quantitative biology.

## Click chemistry in biological studies

Click chemistry does not correspond to one particular chemical reaction. This concept is related to the use of novel chemical reactions, as proposed by Prof. K. Barry Sharpless of the Scripps Research Institute (Kolb et al., 2001). A click chemistry reaction shows the following properties: (1) it uses a solvent that is benign or easily removable, such as water; (2) it only generates inoffensive byproducts; (3) it gives very high chemical yields; (4) it does not need extremely high temperature or pressure; and (5) the products from the reactions are physiologically stable. All of the characteristics above are regarded to be suitable for the chemical labeling of biomolecules, because almost all biomolecules, especially biopolymers, are fragile in the extreme conditions necessary for carrying out the standard chemical reactions. Thus, the number of applications focusing on the use of click chemistry in the life sciences is increasing every year (Best, 2009; Lang and Chin, 2014a).

**Figure 1:**
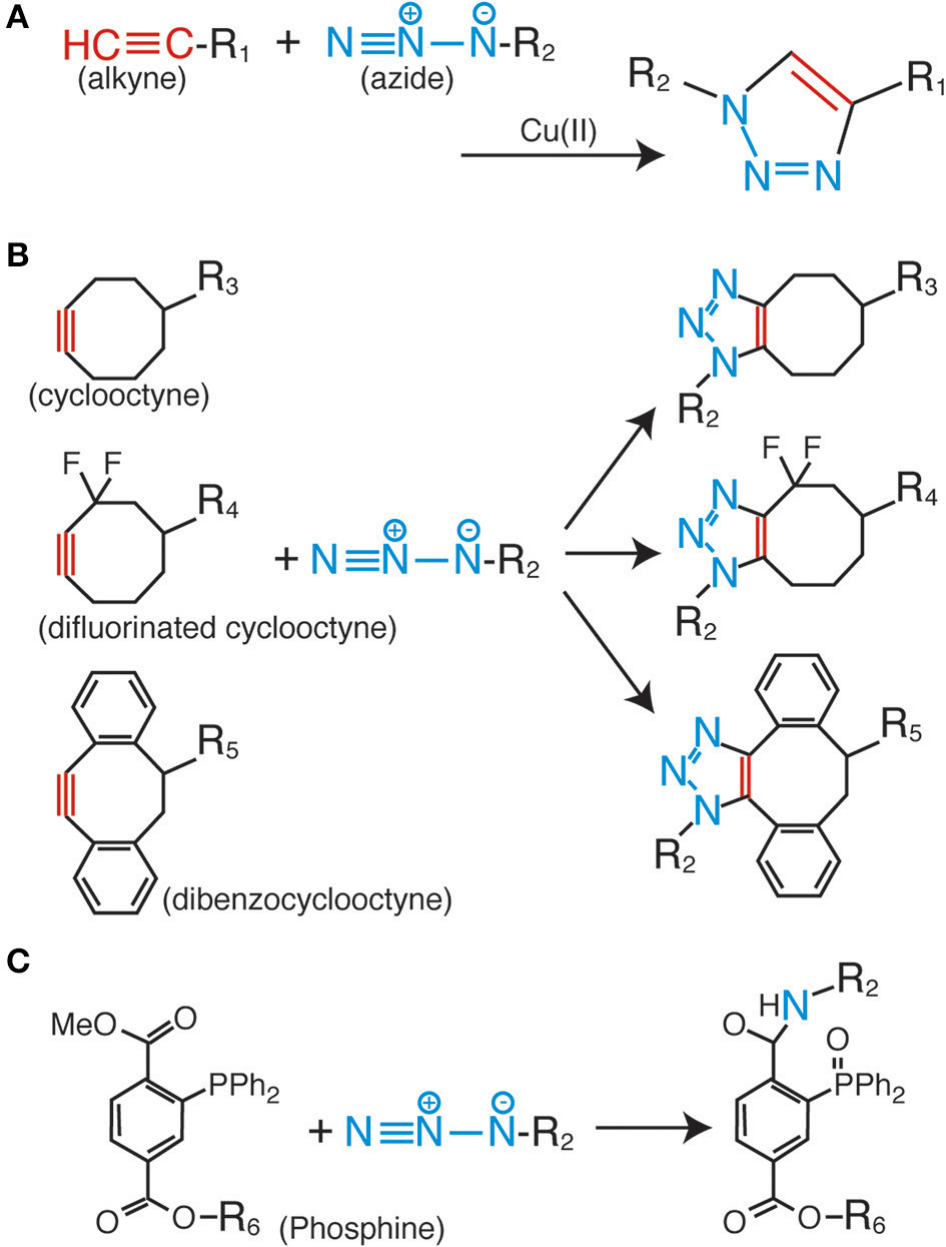
**Huisgen cycloaddition and Staudinger ligation**. **(A)** Copper-catalyzed azide-alkyne cycloaddition (CuAAC). **(B)** Copper-free azide-alkyne cycloaddition reactions using cyclic alkynes. **(C)** Staudinger-Bertozzi ligation.

Among the various click chemistry reactions, the azide-alkyne Huisgen cycloaddition is the most widely employed reaction in biological studies. This is a coupling reaction between the azide and alkyne groups, which form a very stable triazole ring as a linker (Figure 1A) (Huisgen, 1963). For the progression of this reaction, no additional factors are needed, such as heating and high pressure; the only requirement is the use of a monovalent copper ion as a catalyst (Rostovtsev et al., 2002; Tornøe et al., 2002). This reaction is termed the copper-catalyzed azide-alkyne cycloaddition (CuAAC). The most important characteristic of the CuAAC is that the azide and the alkyne do not react with any other molecules inside the cells and tissues. This property enables highly bioorthogonal fluorescent labeling in living and complex samples (Best, 2009; Lang and Chin, 2014a).

Although the fluorescent labeling of various biomolecules such as proteins, peptides, sugar chains, DNAs, RNAs, and lipids via CuAAC has proven to be successful (Lahann, 2009; Lang and Chin, 2014a), several problems still remain to be solved. The most serious issue for CuAAC is the cytotoxicity of the copper ion (Boyce and Bertozzi, 2011). High concentrations of this metal ion catalyst make it impossible to fluorescently label living cells and organisms. As a solution to the problem, copper-free click reactions have been developed in recent years. In these reactions, cyclic derivatives of the alkynyl group [e.g., cyclooctyne (Agard et al., 2004), difluorinated cyclooctyne (Baskin et al., 2007), and dibenzocyclooctyne (Sletten et al., 2010)] are employed as reactive partners for the azide group (Figure 1B). These kinds of cyclic alkyne derivatives harness the intrinsic energy from their distorted structures; therefore, no additional energy or catalysis is needed. Some of these derivatives are already commercially available as fluorescence labeling reagents for living cells, and have been successfully employed in live cell analyses. However, the relatively bulky size of the cyclic alkyl groups affects the membrane permeability of the labeling reagents; therefore, almost all of the previous examples are limited to extracellular labeling studies.

The Staudinger-Bertozzi ligation is regarded as an effective alternative to CuAAC. This chemical reaction also involves a coupling reaction between an azide and a phosphine (Figure 1C) (Saxon and Bertozzi, 2000). Similar to the azide and the alkyne, the phosphine group also does not react with any of the functional groups on the biomolecules. However, the Staudinger-Bertozzi ligation has some useful characteristics for the fluorescent labeling of biomolecules in living cells. Unlike CuAAC, no catalyst is necessary for the coupling reaction between the azide and the phosphine groups. Indeed, several studies showed successful live cell fluorescent imaging with this technology (Chang et al., 2007; Hangauer and Bertozzi, 2008). Although this technology has a potential to be a universal tool for the live cell imaging, the membrane permeability of phosphine-dyes is a remaining issue to be solved.

More recently, novel bioorthogonal uses of “click” reactions have been published (Kodama et al., 2007; Song et al., 2008; Tong et al., 2009; Nguyen et al., 2011). Among them, the most promising reaction for live cell imaging study is the inverse-electron-demand Diels-Alder cycloaddition, which is a coupling reaction between strained alkenes and tetrazines (Blackman et al., 2008). A lot of studies for the live cell imaging by using the reaction have been published in recent years (Selvaraj et al., 2011; Lang et al., 2012; Plass et al., 2012; Liu et al., 2012a). This technology might become a primary tool for live cell imaging studies, because this reaction has not only harmlessnesss for living cells but also a very fast rate constant as compared with other reactions (Lang and Chin, 2014b), whish is an important parameter for the quantitative labeling.

## Induction methods for the anchor groups of biomolecules in living samples

**Figure 2:**
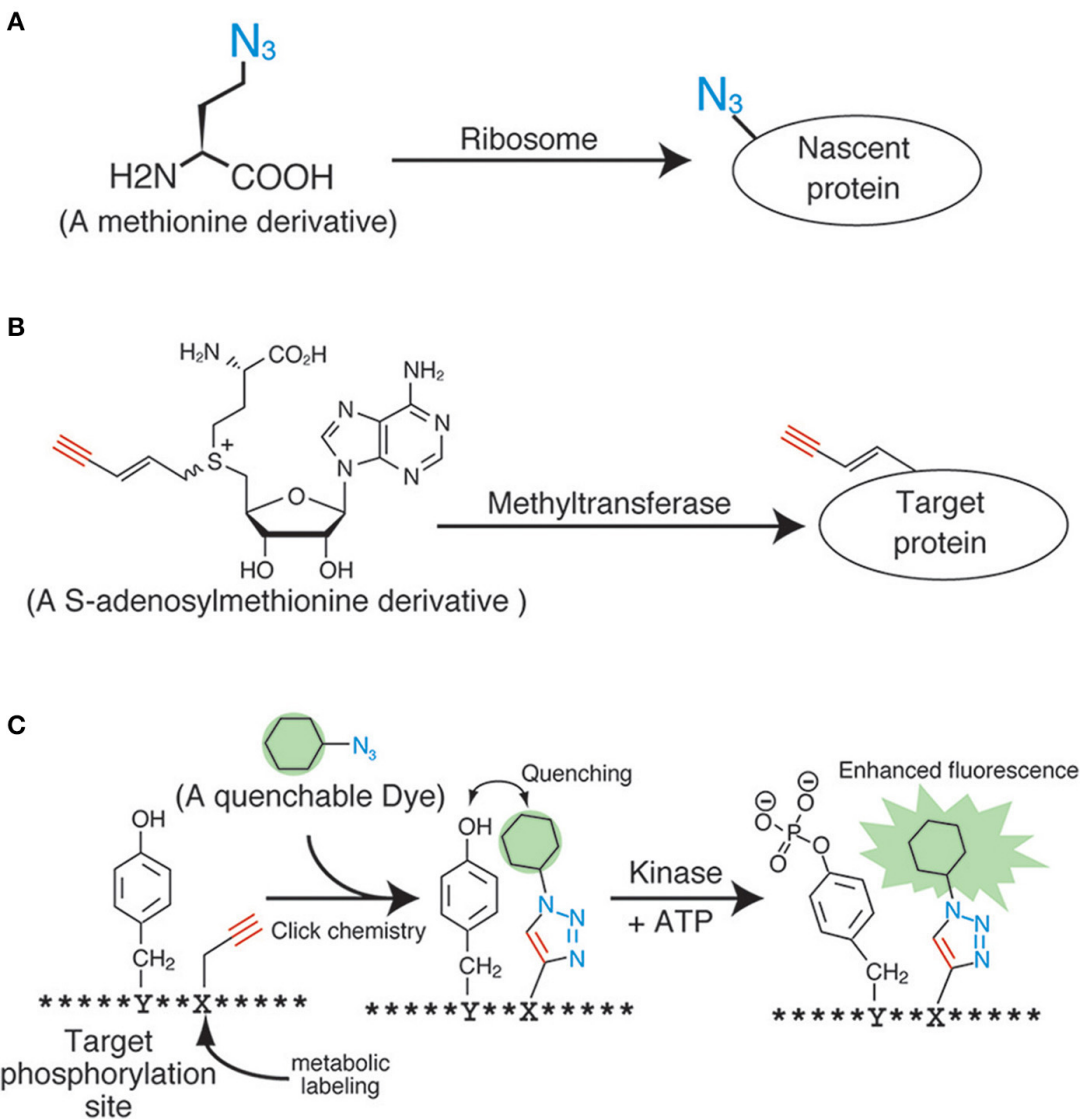
**Applications of click chemistry for biomolecular labeling**. **(A)** An example of the metabolic labeling methods. **(B)** An example of the enzymatic labeling methods. **(C)** A fluorescent chemosensor for detecting phosphorylation.

The application of click chemistry in bioscience is increasing every year. To date, a wide variety of “clickable” reagents for the fluorescent labeling of biomolecules have been identified, many of which are also commercially available. However, the more important and challenging issue pertaining to the fluorescent labeling of living samples with click chemistry is the induction of the anchor groups (i.e., the azide, alkyne, and phosphine groups) into the biomolecules inside living samples. The most widely used induction method for the anchor groups in living cells is through metabolic incorporation, which uses monomer analogs for biopolymers, such as nucleotides, sugars, and amino acids (Figure 2A). These molecular anchors are of a relatively smaller size, thereby making it easier to enzymatically incorporate the derivatives into the biopolymers.

Among the metabolic labeling targets identified for click labeling, the sugar chains on the membrane proteins hold the biggest promise. Although the sugar chains on the membrane proteins have important roles in various physiological phenomena, including cell-cell adhesion, signal transduction, and immunologic response, their specific characteristics such as localization, amount, half-life, function, and the difference between cell types continue to remain elusive. In order to visualize the sugar chains containing particular types of sugars, different types of unnatural monosaccharide residues containing an azide group (azide sugars), i.e., N-azidoacetylmannosamine (Saxon and Bertozzi, 2000), N-azidoacetylglucosamine (Vocadlo et al., 2003), N-azidoacetylgalactosamine (Hang et al., 2003), and 6-azidofucose (Sawa et al., 2006), were produced and used for metabolic labeling studies. These derivatives were successfully incorporated into the sugar chains on the cell membrane through the intrinsic metabolic machinery, and were clearly visualized by following the click ligation with fluorescent dyes. The live imaging of the sugar chains uses this technique, and such experiments have already been done, not only in cultured cells but also in the tissues of living organisms. The copper-free click reagents such as difluorinated cyclooctyne (Figure 1B) have made this possible (Baskin et al., 2007). Therefore, the sugar chains could be regarded as a relatively easier target as compared to the other intracellular biopolymers.

Proteins have also been a primary target for analyses during metabolic labeling and click ligation. Several labeling reagents for newly synthesized proteins have been developed in recent years. Methionine derivatives bearing azido or alkyne groups, termed l-azidehomoalanine and l-homopropagylglycine, respectively (Wang et al., 2008), are the most widely used and commercially available reagents for the metabolic labeling of nascent proteins in living cells (Figure 2A). These are counterparts of the ^35^S-labeled methionine used in radiographic analyses, and are able to label the nascent proteins synthesized during a particular period or under specific signals. Although these methionine analogs are useful and reliable, pretreatment of cells with a methionine-free medium is necessary (Soundrarajan et al., 2012), and this can affect the cells physiologically. Hence, alternatives with different labeling machineries were developed. The derivatives of puromycin, which is an inhibitor of protein biosynthesis, are one of the most promising reagents for the labeling of nascent proteins. The labeling method using these reagents needs no culture condition change, unlike the methionine analogs. Liu et al. reported an alkyne-conjugated puromycin derivative for nascent protein labeling in cultured cells and tissues (Liu et al., 2012b). Beatty and colleagues attempted the live cell imaging of nascent proteins in live fibroblast cells by using the l-azidehomoalanine- and a BODIPY®-conjugated cyclooctyne (Beatty et al., 2011). The fluorescent signals inside the cells were confirmed using confocal microscopy and protein fractionation. However, these data also revealed the preferential click labeling of membrane proteins, which might have resulted from the low cell penetrability of BODIPY®-conjugated cyclooctyne. In order to develop a reliable protein-labeling method for quantitative analyses, the development of membrane-penetrable click reagents seems essential. Genetic code reprograming technologies, which change the usage of codons, are also powerful method to introduce unnatural amino acids into proteins. Several teams have reported a lot of successful result of protein labeling by using the genetic code reprograming (Zhang et al., 2002; Chin et al., 2003).

The biosynthesis of RNA and DNA has also been analyzed in living cells using metabolic labeling followed by click ligation. The data collected from these kinds of analyses can be used to understand DNA replication, DNA repair, and transcriptional control. For example, the use of 5-ethynyl-2′deoxyuridine as a DNA precursor or the use of 5-ethynyluridine as an RNA precursor during microscopic or flow cytometric analyses enables quantitative analyses of DNA replication and whole transcription (Figure 2A) (Darzynkiewicz et al., 2011).

Metabolic labeling coupled with click labeling is a highly effective tool to analyze biomolecular transitions during the cell cycle, cell differentiation, signal responses, and apoptosis. However, the metabolic labeling methods for the anchor groups are not suitable for the analyses of particular species of biomolecules. Therefore, more specific labeling methods will be needed in the future.

## Novel labeling methods and the use of click chemistry in quantitative biology

Several technologies are currently under development for the molecular species-specific labeling of the anchor chemical groups present in biomolecules. The enzymatic labeling method for substrates is one such successful example. Many kinds of biomolecules such as proteins and nucleic acids are modified by methyl groups following neogenesis. These serve as important intrinsic functional markers, and are controlled by various methyltransferases. Prof. E. Weinhold and his colleagues developed analogs of S-adenosylmethionine, a methyl-group donor for almost all of the methyltransferases (Figure 2B) (Klimasauskas and Weinhold, 2007). These analogs work well *in vitro* and *in vivo*, and can modify the substrates with the anchor groups instead of the methyl groups in an enzyme-specific manner. By using this technology, DNAs (Schmidt et al., 2008), RNAs (Motorin et al., 2010), and proteins (Peters et al., 2010) have been successfully labeled with an alkyne or an azide. Similar enzymatic labeling methods for protein substrates have also been studied for other post-translational modifications, including phosphorylation (Lee et al., 2009) and acetylation (Yang et al., 2010). Although these technologies could potentially be used for substrate-specific labeling in living cells, an improvement of the molecular architecture of the methyl-donor analogs is necessary, because the degree of specificity and efficiency is still not up to the mark.

Other novel ideas using click chemistry for biological quantification have also been proposed. For example, Kamaruddin et al. devised a fluorescent chemosensor for detecting tyrosine phosphorylation, by metabolically labeling the residues near the phosphorylation sites with a quenchable dye (Figure 2C) (Kamaruddin et al., 2011). The strategy might enable quantitative phosphorylation analysis in an enzyme-specific manner in living cells.

Novel fluorescent labeling techniques have been continually devised in the past decade. Many more innovative fluorescence labeling methods that use click chemistry are expected to be developed in the near future to enhance research in the field of quantitative biology.

### Conflict of interest statement

The author declares that the research was conducted in the absence of any commercial or financial relationships that could be construed as a potential conflict of interest.
